# The Crucial Role of Cultural and Religious Beliefs on Organ Transplantation

**Published:** 2017-02-01

**Authors:** M. Mostafazadeh-Bora, A. Zarghami

**Affiliations:** 1 *School of Nursing and Midwifery, Isfahan University of Medical Sciences, Isfahan, Iran*; 2 *Department of Neurology, Ayatollah Rohani Hospital, Babol University of Medical Sciences, Babol, Iran*

In a recent issue of the *IJOTM*, there was a special attention to organ donation. In one study, Chakradhar and colleagues, showed an insufficient level of positive attitude and practice among undergraduate dental students in Heyderabad, India [1]. In another article, Oluyombo, *et. al*., indicated disparity of knowledge and willingness regarding organ donation among health care workers in South-West Nigeria [2]. In both studies, there were highly significant differences between religious beliefs and the levels of knowledge and attitudes towards organ donation.

One of the challenging issues for medical team in organ transplantation is the religious and cultural viewpoints towards organ donation among different populations [3]. It is well-known that these beliefs in religious countries, particularly in Asian regions, play a pivotal role on behavior and decision making regarding organ donation [4]. However, little attention and effort has been made to promotion such a positive action. Olutombu and colleagues underlined that there is no law and order against donating organ in religious references. Therefore, it is essential to increase the awareness of population about the principles of organ donation as a life-saving action.

There are numerous examples of positive attitudes towards tissue and organ transplantation in religions. As an example, in The holly *Qur’an* (The holly book of Muslims), it is well-illustrated that removing organs, as the only way of treating the ailment, can be acceptable and the donor and their family members must give the necessary consents to do so. Furthermore, if possible, transplantation should be performed only among Muslims [5].

Altogether, cultural and religious views have important roles in the formation of beliefs about organ donation. By considering diversities in this regards, health professionals are able to react properly. Also, providing an opportunity to consult with a religious leader about organ donation can help families passing through hard circumstances in order to make the best decision.

## Conflicts of Interest:

None declared.
